# Enhancing aerodynamic performance using biomimetic wavy trailing edges on aircraft wing at low Reynolds number

**DOI:** 10.1038/s41598-026-36401-9

**Published:** 2026-02-02

**Authors:** Mohamed A. Aziz, Mohamed A. Khalifa, Haitham Elshimy, Ahmed M. Elsayed

**Affiliations:** 1 Mechanical Engineering Department, Faculty of Engineering, Suez University, P.O.Box: 43221, Suez, Egypt; 2 Mechanical Engineering Department, Institute of Aviation Engineering and Technology, Giza, Egypt; 3 Department of Mechanical and Aerospace Engineering, United Arab Emirates University, Al Ain, United Arab Emirates; 4 Mechanical Engineering Department, Faculty of Engineering, Fayoum University, Fayoum, 63514 Egypt

**Keywords:** Biomimetic design, Wavy trailing edge, Lift enhancement, Stall delay, Numerical simulation, Small aircraft aerodynamics, Engineering, Aerospace engineering, Mechanical engineering

## Abstract

This study investigates the aerodynamic effects of biomimetic wavy trailing edges inspired by natural designs, focusing on their application to a swept-back NACA 0012 airfoil under free-flight conditions. Numerical simulations were conducted using a three-dimensional numerical model with k−ω SST turbulence modeling at a Reynolds number of 3 × 10^4^. The study was performed under steady-state conditions. Reynolds-Averaged Navier–Stokes (RANS) were used in the simulation. The baseline wing was modified with sinusoidal trailing edges, varying wave amplitude and wavelength in both chordwise and spanwise directions to assess their impact on lift, drag, and stall characteristics. Experimental validation was conducted in a low subsonic speed wind tunnel using 3D-printed scaled models, with comprehensive data collection on lift and drag coefficients, pressure distribution, and flow visualization. The results indicate that the wavy trailing-edge configuration maintains comparable lift to the clean wing at low angles of attack (AOA < 8°), while providing substantial aerodynamic enhancement beyond 8°, with improved lift generation and delayed stall. This demonstrates its potential for improving post-stall stability and aerodynamic efficiency in low-Reynolds-number flight regimes. Results demonstrate that a moderate wave amplitude of 20% tip chord length at 8° angle of attack enhances lift by 11.8%. The optimum parametric wavy wing delays the stall by approximate 6° associated with an increase in C_Lmax_ by 31% compared to a conventional straight edge. The findings highlight the potential of wavy trailing edges for improving aerodynamic efficiency and stability, particularly for small aircraft and UAVs.

## Introduction

Nature offers remarkable aerodynamic designs that have inspired engineers to develop efficient and adaptive configurations. Birds’ wings, evolved for various flight regimes, demonstrate optimized aerodynamic performance, flow adaptability, and energy efficiency, which have led to numerous bio-inspired flow control mechanisms^1–3^. These mechanisms, such as stepped or wavy surfaces, have shown potential for stall delay and aerodynamic enhancement at low Reynolds numbers. For instance, stepped airfoils inspired by the multi-layered structure of bird feathers demonstrated improvements in lift coefficient (CL, max) by 11–35% and stall angle delay by 2° compared to conventional airfoils^4,5^.

Flow control methods can generally be categorized into active and passive approaches. Active methods involve external energy input to manipulate flow, while passive methods modify surface geometry to induce favorable flow behavior. Yu et al.^6^ and Nesij et al.^7^ demonstrated that plasma actuators effectively suppress separation and increase lift-to-drag ratio by more than 160%. Similarly, Aziz et al.^8^ experimentally found that pulsating suction jets enhanced lift and reduced drag. Nabhani et al.^9^ employed synthetic jets optimized through genetic algorithms, achieving reattached flow and power improvement of 23–36 kW in turbine airfoils.

In contrast, passive flow control methods inspired by nature have been widely examined for their simplicity and self-sustainability. Fan et al.^10^ investigated humpback whale-inspired leading-edge tubercles, showing improved lift performance and stall stability. Aboelezz et al.^11^ integrated guided vane airfoils in Darrieus turbines, increasing efficiency by 26%. Elsayed et al.^12^ and Aziz and Elsayed^13^ utilized multi-suction and passive air-jet systems, achieving notable aerodynamic improvements under varying flow conditions.

The application of biomimetic morphologies, particularly those inspired by birds and marine life, has yielded distinct aerodynamic advantages such as reduced drag, delayed separation, and enhanced lift^14–16^. Yang et al.^15^ and Rostamzadeh et al.^16^ demonstrated that sinusoidal leading- or trailing-edge configurations enhance aerodynamic efficiency, delay stall, and reduce aerodynamic noise. Yang et al.^17^ further showed that deeper trailing-edge waves reduce drag and noise, while Cai et al.^18^ reported drag reduction exceeding 30% on bluff bodies equipped with sinusoidal trailing edges. Shi et al.^19^ introduced a porous wavy trailing edge that achieved an 8.1 dB noise reduction without sacrificing aerodynamic efficiency.

Recent investigations have further validated these aerodynamic and acoustic benefits. Wang et al.^20^ have discovered the noise drop effects of bio-inspired oblique TE serrations applied to an owl-wing-shaped airfoil at low Mach numbers. Results show that the airfoil with bio-inspired serrations achieves a higher C_L_/C_D_ ratio and significantly reduces noise emissions, with an additional noise reduction achieved using asymmetric serrations. Smith et al.^21^ have examined the use of spanwise wavy airfoils geometries to reduce TE noise and enhance aerodynamic characteristics. Among four variants tested, the most irregular surface, composed of two wavelengths, achieved the highest noise reduction of 17.7 dB and improved drag characteristics by modifying boundary layer dynamics and reducing flow separation. Prigent et al.^22^ have experimentally investigated the wake of a NACA0012 wing with cut-in sinusoidal TEs to understand their impact on flow structures. The sinusoidal modifications reduce vortex shedding intensity by up to 57% compared to a straight blunt wing, while creating spanwise inhomogeneity in the wake’s velocity deficit and width. Maksoud et al.^23^ conducted a study on the aerodynamic performance of modified NACA 634-021 airfoils featuring protuberances on both the LE and TE. Results show that while the standard airfoil performs better at lower angles of attack, the wavy airfoils demonstrate improved post-stall performance and delay stall due to vortex generation. The findings suggest that these protuberances could enhance lift at high angles of attack, making them useful for applications in high-performance aircraft.

Morelli et al.^24^ have numerically explored how sinusoidal (wavy) leading edge designs affect ice accretion on aircraft wings. Results show that shorter wavelengths and larger amplitudes increase droplet impingement, leading to greater ice buildup at wave peaks and troughs, while midsections remain less affected. Chang et al.^25^, have studied the aerodynamic performance of an airfoil with twin leading edge protuberances, inspired by humpback whale fins, to improve stall characteristics. Through wind tunnel experiments, the effects of protuberance amplitude on stall behavior were analyzed. Results show that increasing Reynolds number raises the critical stall angle, and reducing the protuberance amplitude weakens flow control effects while delaying the initial stall onset.

Existing studies tend to focus on specific applications, such as noise reduction in wind turbine blades, without fully leveraging the potential of wavy trailing edges for passive flow control across a broader range of aerodynamic applications, including UAVs and high-performance airfoils. Furthermore, the underlying flow physics associated with wavy trailing edge configurations, particularly in terms of vortex dynamics and boundary layer interactions, remains inadequately explored. This lack of detailed understanding limits the ability to optimize these designs for practical engineering applications.

The current study aims to address this gap by providing a comprehensive investigation into the aerodynamic effects of wavy trailing edge designs. This study investigates the aerodynamic advantages of biomimetic wavy trailing edge designs, with a focus on their potential for passive flow control and performance enhancement. Using computational fluid dynamics simulations and experimental validations, the research explores how varying wave amplitude and wavelength parameters influence aerodynamic efficiency. The findings aim to provide a deeper understanding of the flow physics associated with wavy trailing edges and their applications in sustainable and high-performance engineering designs. Table 1 presents a summary of relevant previous studies from the literature, highlighting their key findings and comparing them to the current work. The term “No. of wavies” refers to number of full sinusoidal cycles distributed along span, meaning that each cycle consists of one peak and one trough.

**Table 1:**
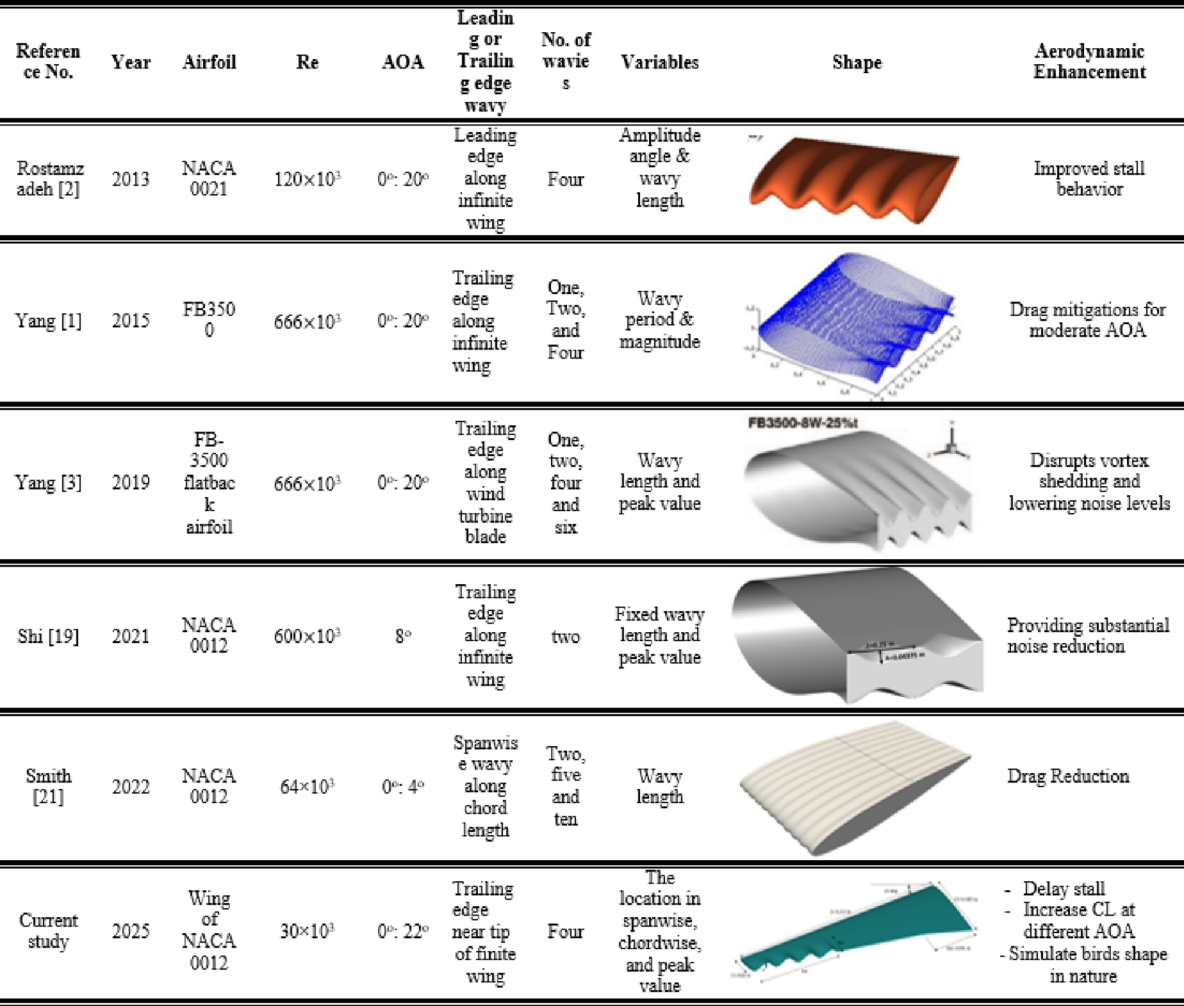
Comparison of previous experimental studies in the literature with the current work.

### Wavy wings inspired from bird trailing edge in nature

**Fig. 1:**
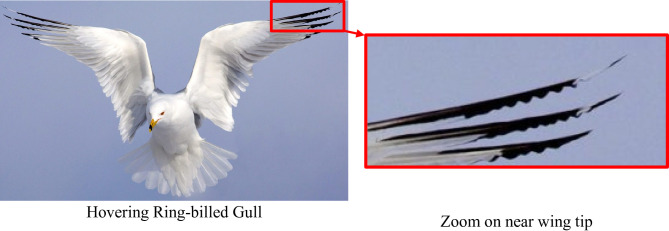
Trailing edge wavy wing shape in nature^26^,

In Fig. 1, a photograph of a Ring-billed Gull hovering in New York, captured by Gerrit Vyn for the Summer 2021 issue of Living Bird magazine. The image was shot using a 300 mm lens, with a shutter speed of 1/2500 seconds at f/8, and ISO 400 to freeze the motion of the gull’s wings in mid-air. The gull’s wing tip and trailing edge display a wavy, undulating structural feature that plays an important role in aerodynamic efficiency and maneuverability. This wavy trailing edge seen in nature often contributes to reduced drag and enhanced lift. The design of the bird’s wing edge suggests passive flow control adaptations that minimize turbulence, enhancing its hovering capability and stability in the air. This observation, illustrated by Fig. 1, highlights how the natural morphology of bird wings, particularly the wavy trailing edges, inspire bio-inspired design in engineering, especially in wing and airfoil designs for MAVs (Micro Air Vehicles) and UAVs.

In the current research, a full numerical and experimental study investigating the effect of geometrical parameters that control the trailing edge (TE) wavy wing tip applied to an aircraft wing would involve detailed analysis of how variations in wave amplitude, wavelength, and wave shape influence the aerodynamic performance of the wing, such as lift, drag, and stall behavior. This research aims at understanding how biomimetic designs inspired by bird wings can improve aircraft performance by improving lift and enhancing overall efficiency and stability. The present study is organized into three main sections. The first section, Computational Setup and Validation, describes the numerical methodology, boundary conditions, and model validation process. The second section, Impact of Wave Parameters, investigates the influence of different wave configurations on aerodynamic characteristics and flow behavior. The third section, Stall Behaviour and Flow Separation, provides an in-depth analysis of the onset of stall and associated flow separation phenomena. The objectives aim to assess the effects of wavy trailing edge geometry on aerodynamic performance, focusing on parameters such as the lift coefficient, C_L_, drag coefficient, C_D_, lift-to-drag ratio, C_L_/C_D_, and stall angle. In addition, optimize wavy geometrical parameters to maximize aerodynamic efficiency and improve stability, especially at various angles of attack, AOA, and compare biomimetic designs inspired by bird wings to conventional straight trailing edge designs to demonstrate potential improvements.

### Numerical analysis

Turbulence is inherently described by irregular, unsteady variations in properties and velocities. Popular modeling approaches include Reynolds-Averaged Navier–Stokes RANS methods^27–29^. In the RANS, any flow field property is decomposed into a temporally averaged, $\bar \emptyset$, and a fluctuating,$\mathop \emptyset \limits^\prime$ components representing turbulence:

1 $$\:\emptyset=\bar \emptyset+\mathop \emptyset \limits^\prime$$

The RANS methodology focuses on solving for the time-averaged quantities while incorporating Reynolds stresses to consider turbulent effects. The simulations were carried out using a steady-state solver. The continuity equation in the RANS formulation is expressed as:

2 $$\:\nabla\:.\left(\rho\:{\bar {\mathrm{V}}}\right)=0$$

The RANS momentum equation takes the form:

3 $$\:\rho\:\frac{D{\bar {\mathrm{V}}}}{Dt}=-\nabla\:{\bar {\mathrm{P}}}+\nabla\:.\left(\mu\:\nabla\:{\bar {\mathrm{V}}}\right)-\nabla\:.\overline {\rho \mathop {\rm{V}}\limits^\prime \mathop {\rm{V}}\limits^\prime }+F$$

where, $\overline {\rho \mathop {{{\mathrm{v}}_{\mathrm{i}}}}\limits^\prime \mathop {{{\mathrm{v}}_{\rm{j}}}}\limits^\prime }$ is the tensor of Reynolds stress representing the momentum exchange due to turbulent fluctuations. To simplify, the Boussinesq hypothesis approximates the Reynolds stress tensor using an eddy viscosity, $\:{\mu\:}_{t}$, as follows:

4 $$\overline {\rho \mathop {{{\mathrm{v}}_{\mathrm{i}}}}\limits^\prime \mathop {{{\mathrm{v}}_{\rm{j}}}}\limits^\prime }={\mu\:}_{t}\left(\frac{\partial\:\overline {{{\mathrm{V}}_{\mathrm{i}}}}}{\partial\:{x}_{j}}+\frac{\partial\:\overline {{{\mathrm{V}}_{\mathrm{i}}}}\overline {{{\mathrm{V}}_{\mathrm{j}}}}}{\partial\:{x}_{i}}\right)-\frac{2}{3}{\uprho\:}k{\delta\:}_{ij}$$

### Geometry and model setup

**Fig. 2:**
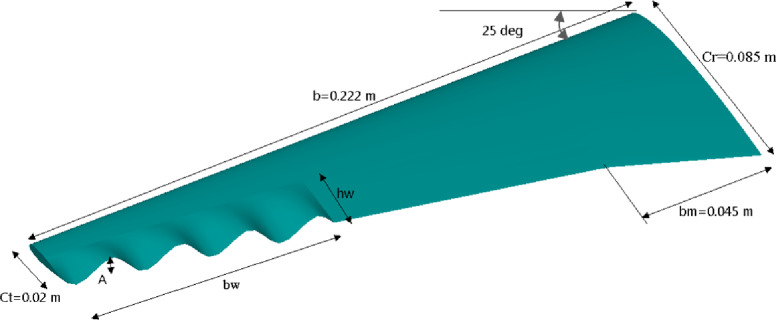
New wing profile geometrical parameter.

The baseline wing geometry was specifically developed for this study to represent a small-scale swept and tapered UAV-type wing based on the NACA 0012 airfoil. This configuration provides a realistic low-Reynolds-number platform suitable for examining the aerodynamic effects of trailing-edge modifications. The root and tip chord length 8.5 cm, 2 cm respectively. The wingspan equals 40 cm with 25° backward sweep as presented in Fig. 2. The trailing edge modifications include varying the wave amplitude defined as distance from peak to trough, wavelength represented by distance between successive peaks. The wave shape is sinusoidal. The sinusoidal trailing-edge geometry is characterized by wave amplitude A, and a wavelength λ, which is the spanwise distance between two successive peaks or troughs. The sinusoidal modulation is applied along the spanwise direction over a section of length b_w_, beginning from the wing tip toward the mid-span. The wave is centered on the local chord line and does not alter the planform shape of the leading edge. Geometric continuity between the wavy and straight trailing-edge regions was ensured using C^1^ continuity, where both the position and slope of the surface were matched at the junction. This configuration introduces spatially periodic undulations along the trailing edge, intended to influence flow characteristics and improve aerodynamic performance. The analytic definition of the wavy trailing-edge profile has been defined as:

5 $$\:{Y}_{T.E}\left(Z\right)=A\times\:\mathrm{s}\mathrm{i}\mathrm{n}(\frac{2\pi\:Z}{\lambda\:}$$

where A represents the wave amplitude and λ is the wavelength along the spanwise (z) direction. In this study, A refers to the full amplitude (i.e., the total vertical displacement from crest to trough is equal to A).

The geometry of the baseline and wavy trailing-edge wings was generated using the CFD-GEOM module within the CFDRC package. The sinusoidal trailing-edge modification was parametrically defined by specifying the wave amplitude (A) and wavelength (λ) along the spanwise direction, applied over the selected portion of the trailing edge from the tip toward mid-span. Creating the geometry within the CFD environment ensured accurate dimensional control and consistency among all simulated cases.

### Computational domain

**Fig. 3:**
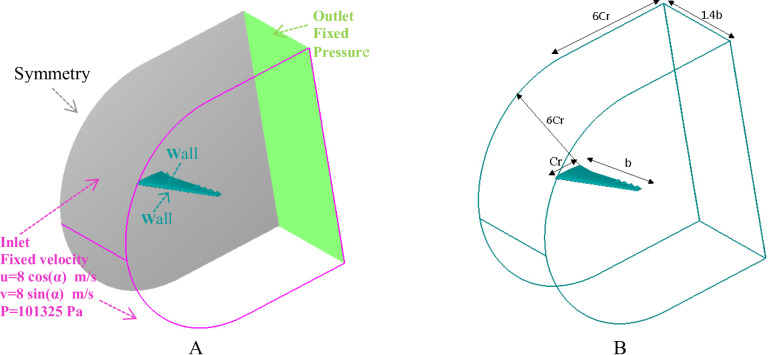
(**a**) Boundary conditions (**b**) Control volume.

A domain width of 1.4b was selected to match the test section geometry of the wind tunnel used for experimental validation and to ensure consistent boundary interactions between the numerical and physical setups. To ensure that the lateral computational boundary located at 1.4b from the wing tip (see Fig. 3), does not influence the flow field or the wing-tip vortex, a domain-sensitivity study was performed. A representative case (NACA0012, Re = 3 × 10^4^, A = 20%C_t_ (0.004 m), λ = 0.25%b_w_ (0.02655), α = 10 degrees) was recomputed using two larger spanwise domain sizes: (i) lateral wall at 2.5b and (ii) lateral wall at 4.0b from the wing tip. All other numerical settings were kept identical. The meshes for the extended domains preserved the same wall-normal and chordwise resolution near the wing; only the spanwise extent and the mesh between wing and lateral wall were increased. Table 2 summarizes the differences in *C*_*L*_ and *C*_*D*_ between the baseline domain (1.4b) and the extended domains. The extended domains produced variations in of less than 1% and no systematic change in the aerodynamic coefficient values beyond the expected numerical uncertainty. Therefore, the lateral wall at 1.4b does not materially influence the flow features discussed in this study, and the reported aerodynamic effects of the wavy trailing edge are robust with respect to domain extent.

**Table 2:** Effect of lateral distance on aerodynamics characteristics.

Domain lateral distance from tip	C_L_	∆C_L_vs. 1.4b (%)	C_D_	∆C_D_vs. 1.4b (%)
1.4 b (baseline)	0.741	–	0.036	–
2.5 b	0.744	+ 0.4%	0.0357	− 0.6%
4.0 b	0.7395	− 0.2%	0.0361	+ 0.3%

In this study, a 3D CFD domain was created to simulate the wing’s aerodynamic performance under free-flight conditions. Figure 3 presents the domain includes a detailed wing profile with boundary conditions designed to replicate a typical flight scenario. At the inlet boundary, a uniform flow velocity of 8 m/s was applied, which corresponds to a Reynolds number of approximately 3 × 10^4^ based on the average wing’s chord length. These correspond to Mach numbers M ≈ 0.024. Using an isentropic small-Mach approximation (Δρ/ρ ≈ 1/2M^2^), the maximum density variation is ≲ 0.03%. Because M≪0.3 and density changes are well below 1%, the incompressible flow assumption used in this work is justified.

The Reynolds number was calculated as the product of air density, flow velocity, and average (mean) chord length, divided by the dynamic viscosity. This velocity and Reynolds-number range is realistic for small unmanned aerial vehicles and micro-air vehicles, which often fly at chord-based Reynolds numbers on the order of 10^4^–10^5^ or slightly above^30,31^, ensuring that the flow conditions reflect actual operational settings. The outlet boundary was set to a pressure outlet with ambient pressure conditions to allow for unrestricted flow out of the domain, effectively simulating the unbounded nature of airflow beyond the wing in flight. This setup enables the simulation to capture aerodynamic effects such as wake formation and flow separation accurately, providing insights into the wing’s performance across varying conditions without artificial constraints on the airflow.

To verify that these extents produce negligible outlet contamination, we monitored the RMS velocity and static-pressure perturbations on the outlet plane and compared them to free-stream values. The maximum velocity perturbation on the outlet plane was found to be < 0.8% of U ∞ and the maximum static pressure deviation was < 0.6% of the free-stream stagnation pressure, both well below the 1% threshold.

The Reynolds number in the numerical simulations was matched exactly to the experimental value of 3 × 10^4^ to maintain dynamic similarity between both approaches. This matching ensured that the flow regimes and aerodynamic characteristics remained consistent, allowing direct comparison and validation of the numerical results against the experimental measurements.

The numerical simulations were performed using the CFDRC commercial CFD package (CFD Research Corporation). This solver employs a finite-volume formulation with a pressure-based segregated algorithm to solve the Navier–Stokes equations. Second-order spatial discretization was applied for all transport equations, and temporal discretization used a second-order implicit scheme. The k–ω SST turbulence model was adopted to capture the transitional characteristics of the low Reynolds number flow (Re = 3 × 10^4^). The CFDRC package was selected for its validated performance in low-Reynolds-number aerodynamic simulations, its robust convergence behavior, and its reliable steady-state solver capabilities for external aerodynamic flows.

The Shear Stress Transport (SST) k–ω turbulence model implemented in the CFDRC solver follows the formulation of Menter^32^, which blends the k–ε model in the free stream and the k–ω model near the wall to ensure accurate prediction of adverse pressure gradients and flow separation. The model constants used are (β* = 0.09, σk = 0.85, σω = 0.5, and a_1_ = 0.31).

### Meshing and grid independent study

**Fig. 4:**
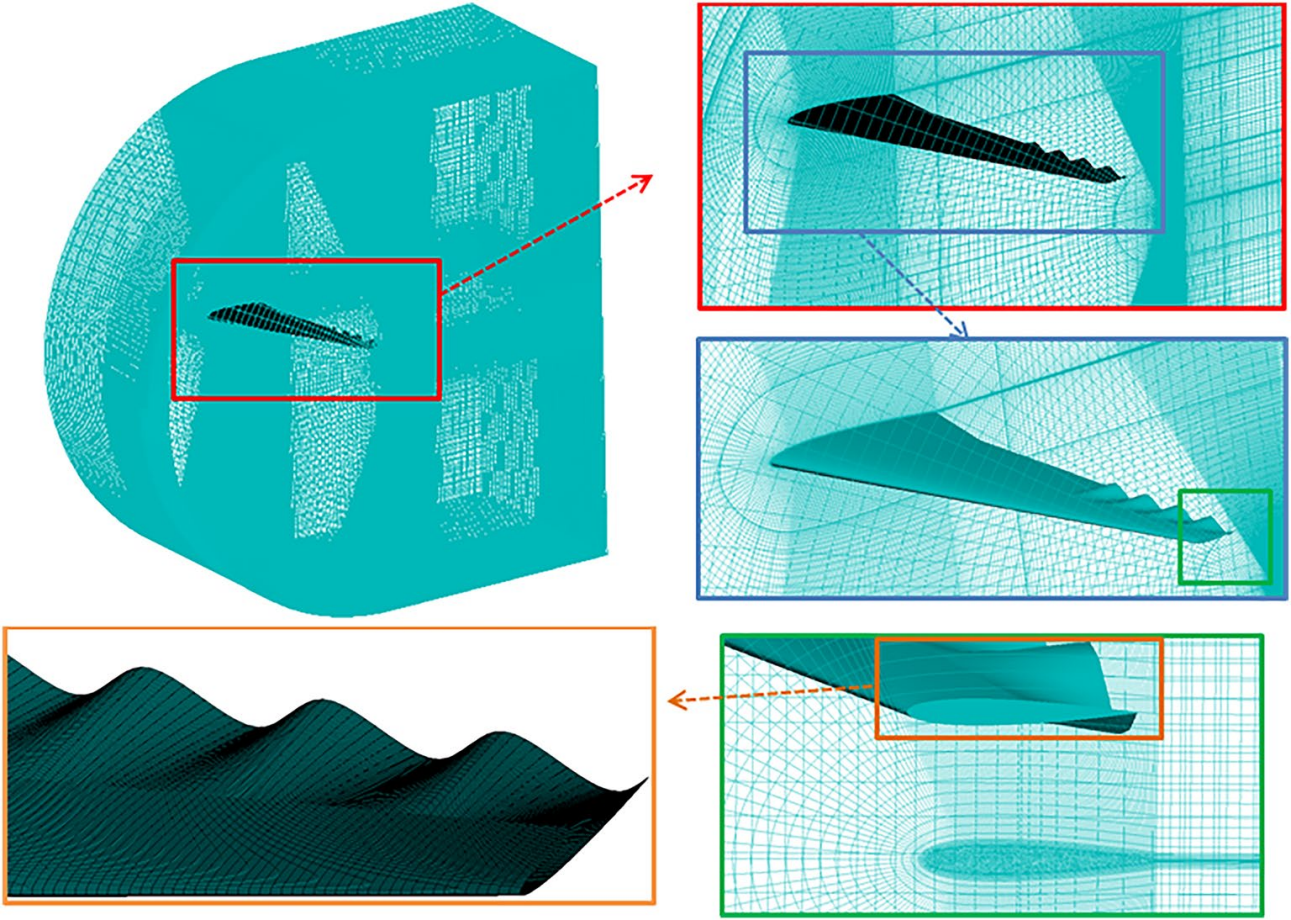
Wavy wing structure grid and zoom on different locations.

A high-quality, structured mesh was employed around the wing profile to capture detailed aerodynamic phenomena, especially in critical regions like the trailing edge, Fig. 4. The mesh consists of approximately 2,300,000 cells, strategically refined near the wing surface and trailing edge to improve accuracy where flow separation and turbulence are anticipated. A y + value of 1 was targeted near the wall to ensure proper resolution within the boundary layer. The near-wall mesh was refined to achieve y + ≈ 1, ensuring full resolution of the viscous sublayer without employing wall functions. This approach allowed accurate prediction of boundary-layer behavior and flow separation characteristics under low-Reynolds-number conditions. The mesh density increased around the trailing edge, with cell sizes as fine as 0.01 mm to capture intricate vortex shedding and separation effects. In the far field, the mesh was gradually coarsened, ensuring computational efficiency without sacrificing accuracy near the wing. This structured approach provided a detailed flow field while maintaining computational stability and efficiency, essential for accurate simulation of lift, drag, and other aerodynamic forces acting on the wing. Each sinusoidal wavelength of the wavy trailing edge was resolved by approximately 120–150 cells in the spanwise direction, ensuring accurate capture of wave induced flow features and vortex dynamics. The nondimensional grid spacing values were maintained within the following ranges: Δx^+^ = 30–60, Δy^+^ = 0.8–1.2, Δz^+^ = 25–50. These settings satisfy the turbulence model’s recommended near-wall requirements Menter^32^ and provide sufficient grid density to accurately resolve both the boundary layer evolution and vortex shedding mechanisms induced by the wavy trailing edge.

For this simulation, the k−ω SST (Shear Stress Transport) turbulence model was selected due to its effectiveness in accurately capturing flow separation and near-wall effects, both of which are critical in aerodynamic analyses of wings. The k−ω SST model combines the benefits of both the k−ω model near the wall and the k−ε model away from the wall, allowing it to predict flow separation more accurately than many other turbulence models. A turbulent intensity equals 5% at the inlet, representative of moderate turbulence in free-flight conditions. The inlet turbulence length scale used in the simulations was specified as a fraction of the local chord, not an absolute 0.1 mm. Concretely, the inlet integral length scale was set to 0.1c (10% of the reference chord), which corresponds to 8.5 mm for the root chord (c = 85 mm) and 2.0 mm for the tip chord (c = 20 mm). This model choice ensures high accuracy in capturing adverse pressure gradients and separation zones, particularly around the trailing edge, which is essential for realistic predictions of lift and drag forces. The SIMPLE algorithm was employed for pressure-velocity coupling, an effective approach for achieving convergence in incompressible flow simulations. The pressure–velocity coupling was handled using the SIMPLE algorithm, which ensures stable convergence for steady-state incompressible flow problems. The convective terms were discretized using a second-order upwind scheme, while the pressure term was treated. Diffusion terms were discretized using second-order central differencing. These settings collectively ensured second-order accuracy and numerical stability throughout the computations. The SIMPLE method iteratively adjusts the pressure field to ensure mass continuity while simultaneously solving for velocity, making it well-suited to steady flow conditions around the wing. The relaxation factors were set at 0.3 for pressure and 0.7 for velocity, balancing stability and convergence speed for this low Reynolds number flow. The under-relaxation factors allow smoother adjustments in each iteration, preventing divergence and enabling the solution to reach an accurate steady state efficiently. This coupling method was selected for its robustness and efficiency in handling aerodynamic flows, ensuring that the pressure and velocity fields accurately represent realistic flight conditions, particularly in regions with complex flow phenomena, such as the trailing edge.

**Fig. 5:**
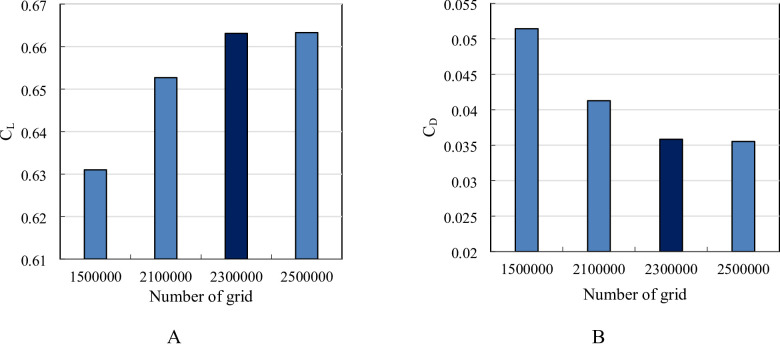
Total number of grid versus (**A**) lift coefficient and (**B**) drag coefficient.

**Fig. 6:**
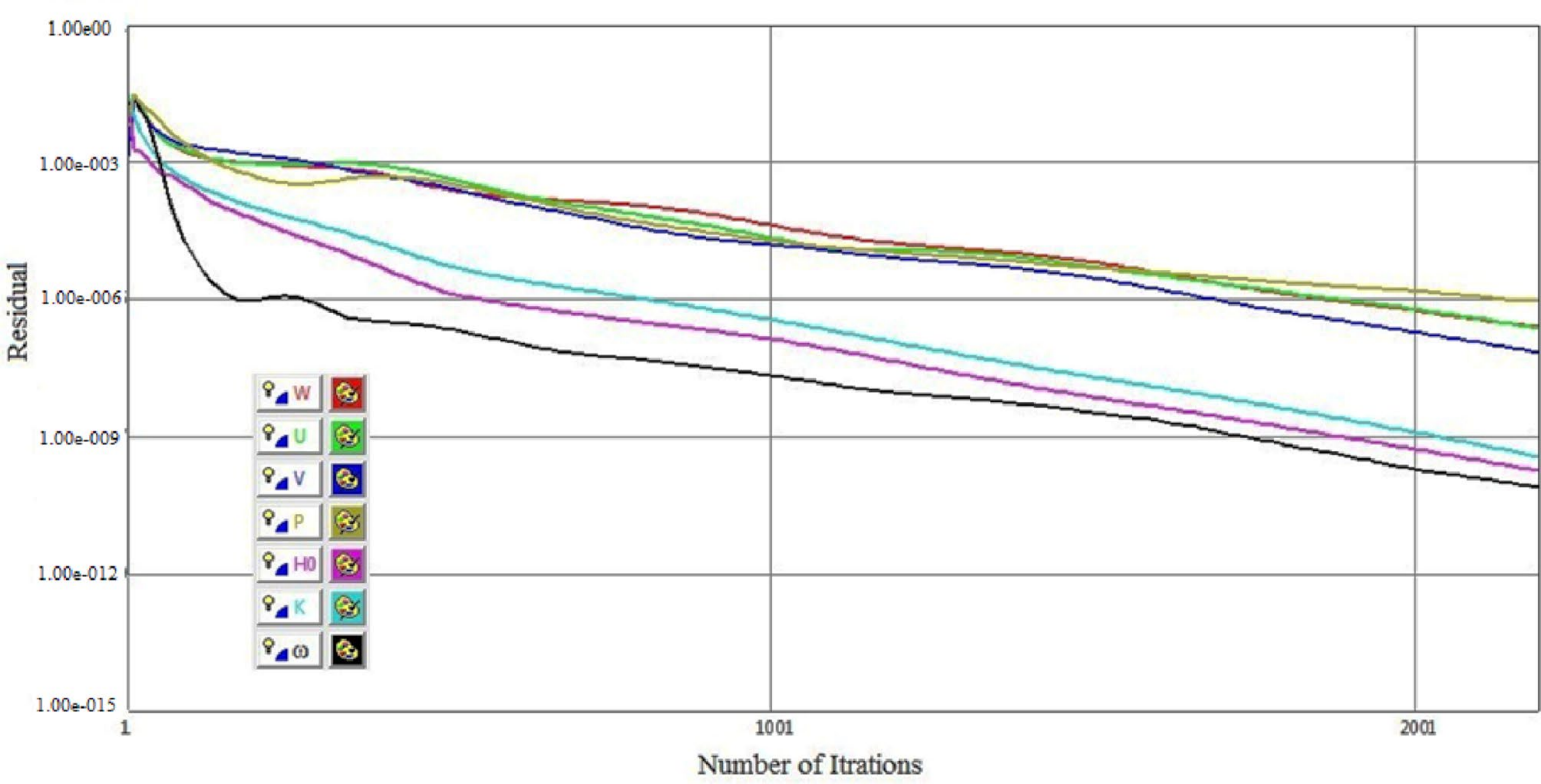
Convergence histories of residuals for velocity components (U, V, W), pressure (P), turbulence kinetic energy (k), specific dissipation rate (ω), and total enthalpy (Ho).

The grid independence study for a three-dimensional wing simulation using CFD demonstrates the impact of grid refinement on the computed C_L_ and C_D_ as presented in Fig. 5. As the number of grid points increases from 1.5 million to 2.3 million, there is a noticeable rise in C_L_ and decrease in C_D_, indicating that the initial coarse grids were insufficient to capture the aerodynamic forces accurately. The highest C_L_ and Lowest C_D_ is observed at 2.3 million grid points, suggesting that this resolution provides a more accurate prediction of lift. However, further refinement to 2.5 million grid points results in only a marginal change in C_L_ and C_D_, signifying that the solution has nearly converged and additional grid refinement does not significantly improve accuracy. The Grid Convergence Index (GCI) was calculated based on the method of Celik et al.^33^, yielding GCI_fine_ = 0.42% for *C*_*L*_ and 0.65% for *C*_*D*_. Accordingly, the mesh with 2.3 million cells was selected for all subsequent simulations as a compromise between accuracy and computational efficiency. The convergence histories for all monitored parameters have been provided in Fig. 6. The numerical simulations were iterated until the residuals of all governing equations (continuity, momentum, turbulence kinetic energy k, and specific dissipation rate ω) dropped below 10^− 6^.

## Experimental analysis

### Model fabrication

**Fig. 7:**
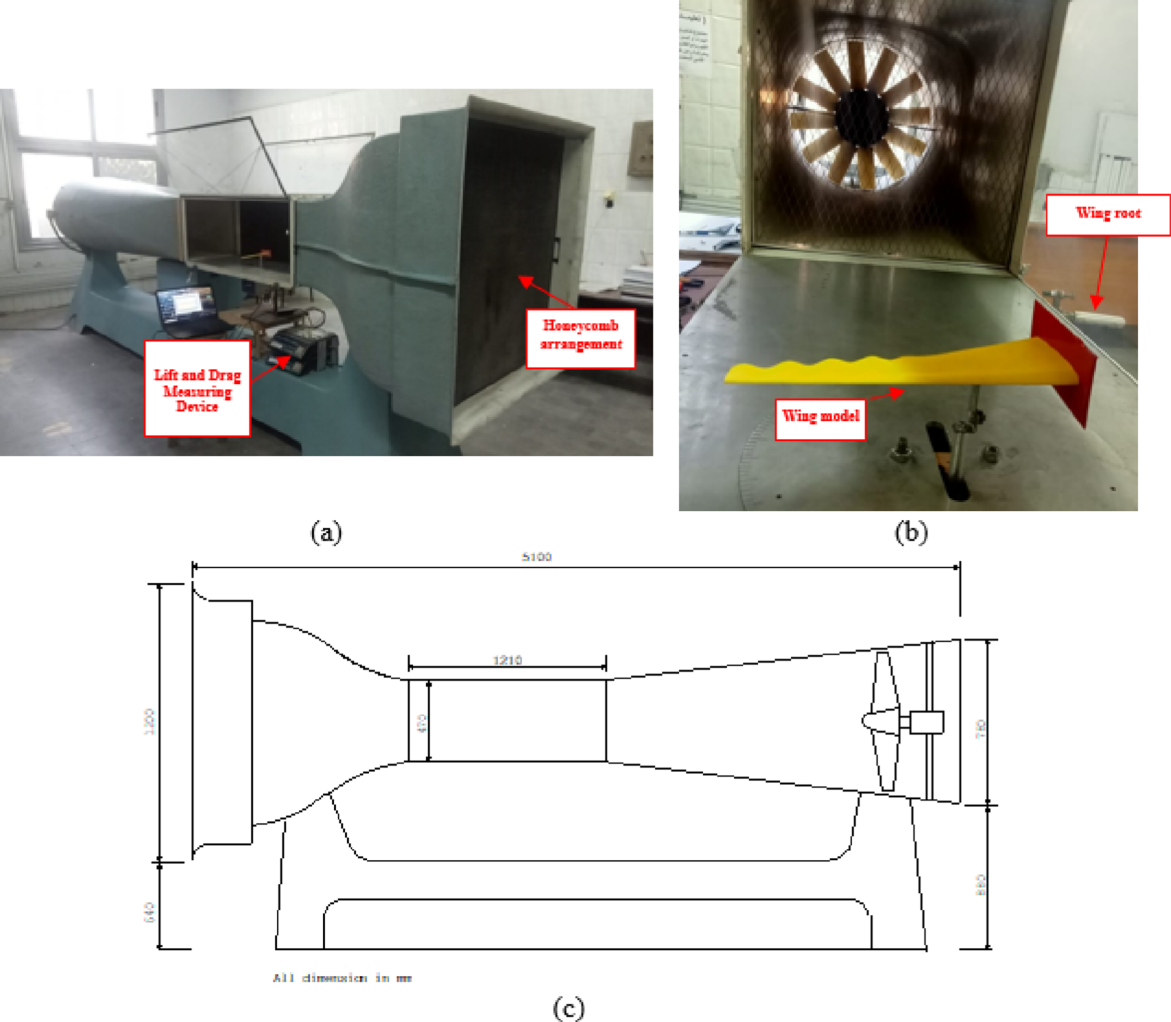
(**a**) Variable-speed wind tunnel, (**b**) test section with T.E. wavy wing model, and (**c**) main dimensions of wind tunnel.

The empirical assessment of both the clean and modified wavy wings was carried out. All experiments were conducted at the Institute of Aeronautical Engineering and Technology (IAET), Aero-Lab. The test section of an open-circuit wind tunnel measuring 47 cm × 47 cm × 121 cm, as shown in Fig. 7. The wind tunnel includes a flow-conditioning section composed of a honeycomb straightener and multiple fine mesh screens located upstream of the contraction zone. This setup ensures a uniform velocity profile and reduces turbulence intensity before the flow enters the test section. Figure 7a shows the installed honeycomb structure, while Fig. 7b presents the test model mounted inside the test section and the rear part of the tunnel, indicating the flow direction and fan arrangement. A scaled-down wing models with wavy trailing edges in a physical lab environment was created. The models based on the wave amplitude, wavelength, and shape used in the numerical study. The model dimensions were selected to maintain a blockage ratio of approximately 4.3%, well within the acceptable range (< 5%) for aerodynamic testing to ensure negligible wall effects and uniform flow distribution.

To ensure the accuracy of the manufactured wavy trailing-edge geometries, each 3D-printed model was inspected using a digital vernier caliper and optical surface measurement to verify dimensional fidelity. The achieved manufacturing tolerance was within ± 0.1 mm, corresponding to less than 2.5% of the wave amplitude (A = 0.004 m) and 1% of the wavelength (λ = 0.04 m). This high level of precision confirms that the printed models accurately reproduced the designed wavy contours, minimizing geometric uncertainty and ensuring the reliability of the aerodynamic measurements.

To ensure reliable results, the wind tunnel tests required minimizing turbulence characteristics. Turbulence intensity was measured using hot-wire anemometry, revealing a value of approximately 1.5% and a turbulence length scale of (order 1–10 mm) in the test section. Careful control and measurement of these turbulence parameters improved the precision and reproducibility of aerodynamic measurements. Each experimental test was conducted three times to assess the repeatability of the lift coefficient measurements. The variation among repeated runs was found to be minimal, with a standard deviation of less than 1.2%, confirming that the aerodynamic data are highly repeatable and that the testing setup provided stable and reliable results across all configurations.

### Material and accuracy

To ensure the trailing edge details are captured accurately, a 3D-printed ABS material suitable for wind tunnel testing was used. The precision of experimental results largely relies on the accuracy of measurement instruments, making it essential to analyze errors in the experimental outcomes. To achieve this, a standardized analysis method proposed by^34^ is applied, as shown in Eqs. (6) and (7).

6 $$\:P=P({x}_{1}+{x}_{2}+\dots\:{x}_{n})$$

7 $$\:{U}_{R}=\sqrt{\sum\:_{i=1}^{n}{\left(\frac{\partial\:P}{\partial\:{x}_{i}}{U}_{i}\right)}^{2}}$$

In the current experiments, the uncertainty analysis for the derived parameters indicates an uncertainty of 1.8% for the Reynolds number (Re) and 5.4% for both the lift coefficient (CL) and the drag coefficient (CD).

**Fig. 8:**
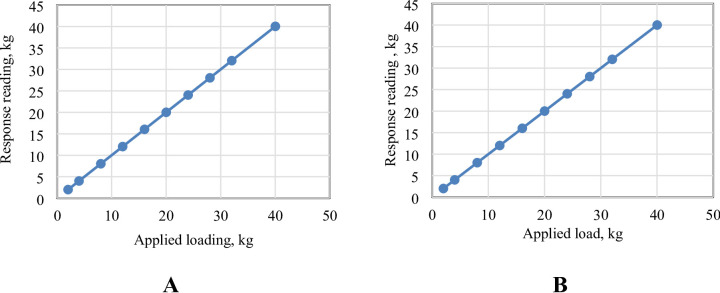
The responses reading versus applied load for **A** lift force load cell and **B** drag force load cell.

The aerodynamic forces measuring device (load cell) was calibrated at the National Institute for Standards (NIS) in Egypt. The statistical analysis showed that there is an average error in measurements that does not exceed 0.76% in both cells. Figure 8 shows the variation in measurement with the change in the standard weights of both cells, which shows the same percentage of error.

The angle of attack (α) was adjusted using a precision rotational stage integrated with a digital inclinometer of 0.1° resolution. Fine adjustment screws were used to achieve the desired angular positions, which were verified prior to each test run. The total uncertainty in the angle-of-attack setting, including mechanical backlash and measurement precision, was estimated to be within ± 0.2°, ensuring accurate and repeatable aerodynamic force measurements.

**Fig. 9:**
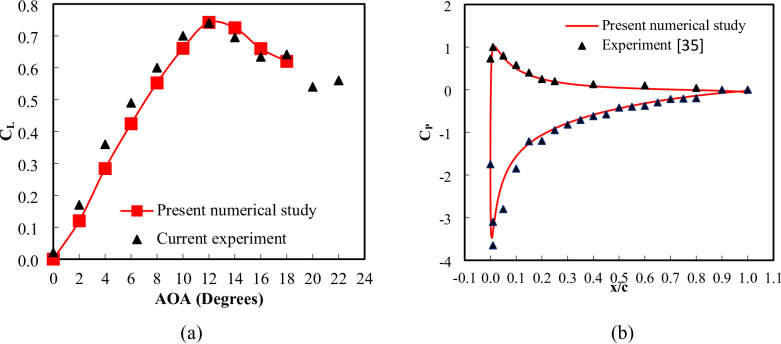
(**a**) numerical and measured lift coefficient for a three-dimensional wing across various angles of attack (**b**) 2D coefficient of pressure with measurements by^35^ at AOA = 10°.

The validation curve compares the C_L_ obtained from the numerical simulation of a three-dimensional wing with experimental measurements across different AOA is shown in Fig. 9a. The term ‘current experiment’ refers to the experimental measurements obtained in the present work. The comparison shows close agreement between the numerical and experimental results, confirming that the numerical model accurately captures the lift slope and stall angle behavior of the airfoil at low Reynolds number. A detailed error analysis reveals that the percentage error fluctuates, with the highest deviation occurring at AOA = 2° (14.29%), indicating a slight underprediction of lift at low AOAs, while the lowest error of 1.27% is observed at AOA = 14°, demonstrating strong agreement near peak lift conditions. The root mean square error between the numerical and experimental C_L_ values is calculated to be 0.0258 for AOA range 0 to 18 degrees corresponding to a normalized RMSE of 1.95% relative to C_L, max_ = 0.74, which suggests a relatively low overall discrepancy and confirms that the numerical approach provides a reliable approximation of the experimental data. The overall closeness between the numerical and measured data results is strong, affirming the validity of the computational model for predicting the aerodynamic performance of the three-dimensional wing within the tested range. Figure 9 (b) presents a validation comparison between the present numerical study and experimental data from^35^ for a two-dimensional NACA 0012 airfoil. The closeness between the numerical and measured data is generally strong, particularly in the mid-chord and trailing-edge regions, where the pressure distributions closely follow the experimental trend. The RMSE between the two datasets provides a quantitative measure of the deviation, indicating the overall accuracy of the numerical approach. The numerical model successfully captures the aerodynamic characteristics of the NACA airfoil, demonstrating its validity for further aerodynamic investigations.

### Impact of wave parameters

All numerical simulations of the parametric study were conducted at a moderate angle of attack of 8°, as it represents the condition near the onset of flow separation, where aerodynamic performance and flow-control effects become more pronounced. This angle allows for a clear assessment of the influence of the wavy wall design on delaying flow separation and enhancing lift. Table 3 summarizes the nondimensional parameters considered in the present study. This approach enables a direct evaluation of how varying the parameters influence the aerodynamic performance, particularly lift enhancement and stall delay, under identical flow conditions.

**Table 3:** Nondimensional parameters used in the present study.

Parameter	Definition	Value/range	Status
Re	Reynolds number	3 × 10^4^	Constant
A/c_t_	Wave amplitude-to-tip chord ratio	0.05, 0.15, 0.25	Varied
b_w_/ (b-bm)	Wavy span ratio	0.3, 0.4, 0.5, 0.6 and 0.7	Varied
h_w_ /c	Wavelength in chord direction-to-chord ratio	0.3, 0.4, 0.5 and 0.6	Varied
Λ	Sweep back angle	25°	Constant
AR	Aspect ratio	4.5	Constant

#### Effect of wave amplitude

In assessing the impact of wave amplitude on aerodynamic characteristics, it was found that moderate wave amplitudes along the trailing edge could enhance lift by improving pressure on wing lower surface, flow attachment and reducing wake formation, while excessively large amplitudes led to higher pressure associated with high drag.

**Fig. 10:**
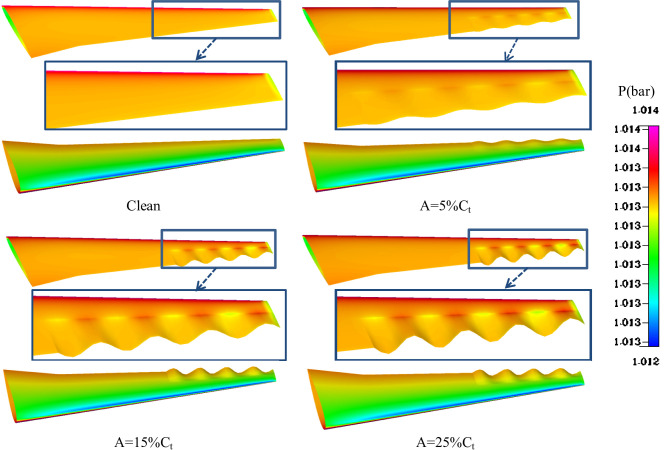
Effect of wavy peak on pressure contours at h_w_ = 50%C and b_w_ = 50%(b-bm).

Figure 10 illustrates the effect of varying amplitudes of wavy peaks on the pressure contours of an airfoil. The results for four configurations; clean airfoil and wavy airfoils with amplitudes 5, 15 and 25%C_t_ are analyzed to demonstrate the aerodynamic improvements brought by the wavy TE. In the clean wing configuration, the pressure contours show a relatively uniform distribution along the span. The transition from high to low pressure is sharp, this results in limited pressure recovery and the potential for increased wake turbulence downstream. For the wing with a wavy TE of small amplitude A = 5%C_t_, localized pressure variations begin to emerge. The contours reveal smoother transitions between high- and low-pressure regions, indicating that the waves disrupt adverse pressure gradients. This disruption reduces the likelihood of premature flow separation and enhances flow stability. The design promotes energy efficient flow behavior, with improved wake structure and reduced drag. When the amplitude is increased to 15%C_t_, the pressure variations become more pronounced. The peaks of the waves generate high-pressure zones, while the troughs create extended low-pressure regions. This configuration demonstrates a stronger ability to control wake turbulence and improve pressure recovery close to the TE. The enhanced flow mixing contributes to superior aerodynamic performance, particularly in reducing drag and increasing lift, making this amplitude particularly effective for practical applications. For the largest amplitude A = 25%C_t_, the pressure recovery close to the TE is further improved. The pronounced wavy geometry promotes significant mixing of high- and low-pressure regions, leading to a smaller wake and reduced drag.

As shown in Fig. 10, the surface pressure near the wing tip exhibits noticeable gradients associated with the generation of the tip vortex. For the clean configuration, a sharp pressure drop occurs at the upper trailing-edge region near the tip, indicating strong vortex-induced suction. In contrast, the wavy trailing-edge configurations modify this behavior by introducing localized pressure modulations that weaken and redistribute the vortex core strength. At moderate wave amplitudes (A = 15–20% Ct), the pressure recovery near the tip is smoother, suggesting partial suppression of the intense suction peak responsible for strong vortex roll-up. This effect contributes to improved flow attachment and reduced induced drag. However, for larger amplitudes (A = 25% Ct), the wave-induced disturbances enhance three-dimensional pressure variations near the tip, promoting additional small-scale vortical structures. Overall, the wavy trailing edge acts to modulate the tip vortex intensity and improve flow stability, particularly at higher angles of attack.

**Fig. 11:**
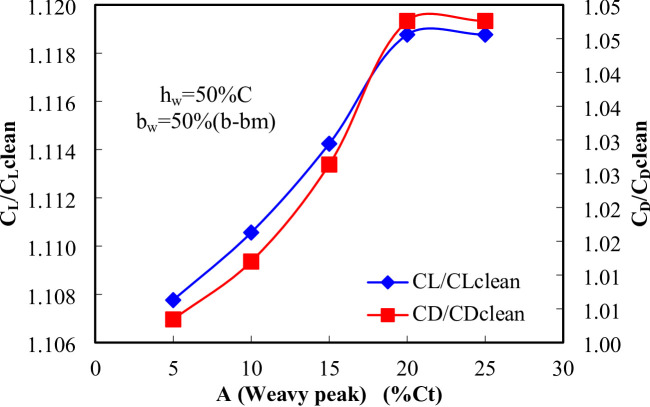
Effect of wavy peak on aerodynamics coefficients at h_w_ = 50%C and b_w_ = 50%(b-bm).

Figure 11 shows testing amplitudes from 5% to 25% of the tip chord length, it was observed that at a moderate amplitude of 20% Ct, the CL increased by 11.8% in compare with the clean wing, with a minimal CD increment of 4.86%. This moderate amplitude effectively disrupted the wake without significantly enlarging it, resulting in improved overall lift-to-drag ratios. However, as amplitude increased to 25% of the chord length and beyond, the drag coefficient rose sharply by approximately 4.7%, as the larger waves intensified flow separation, leading to greater turbulence and energy losses in the wake. This analysis demonstrates that moderate wave amplitudes are optimal for balancing enhanced lift with controlled drag, whereas amplitudes that are too large may negatively impact aerodynamic efficiency by introducing excessive drag.

The amplitude range investigated in this study varied from 5% to 25% of the tip chord length (Ct) to assess its influence on aerodynamic performance. Among these cases, the configuration with an amplitude of 20% Ct (A = 0.004 m) exhibited the highest lift coefficient, indicating an optimal wavy geometry for enhanced aerodynamic efficiency.

#### Effect of wavelength in chordwise direction (h_w_)

**Fig. 12:**
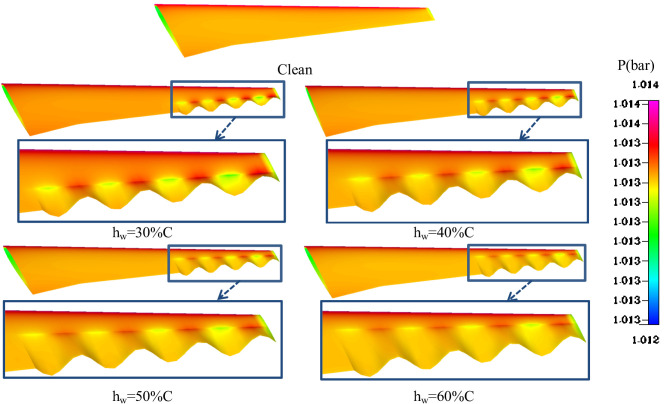
Effect of wavy length in chordwise direction (h_w_) on pressure contours at A = 0.004 m and b_w_=50%(b-bm).

Figure 12 presents the analysis of pressure contours for various wavy configurations along the trailing edge revealing distinct aerodynamic effects compared to the clean configuration. The clean airfoil, with a smooth trailing edge, exhibits consistent pressure distribution, but lacks the flow manipulation necessary for enhanced aerodynamic efficiency. The introduction of wavy structures starting with hw = 30%C begins to influence the pressure distribution. Localized high-pressure regions form near the wave crests, promoting flow stability by delaying separation. As the height increases to hw = 40%C, the pressure gradients become more pronounced, with clearer separation between high- and low-pressure zones, enhancing the mixing and control of the trailing edge flow. The configuration with hw = 50%C demonstrates the most balanced aerodynamic performance. It achieves optimal pressure redistribution by enhancing flow attachment and minimizing flow separation while maintaining manageable drag levels. This configuration ensures smoother transitions between the trailing edge pressure zones, as evident by the well-distributed high-pressure regions (red zones) and consistent low-pressure troughs (blue zones). This balance effectively improves lift generation and reduces drag penalties.

**Fig. 13:**
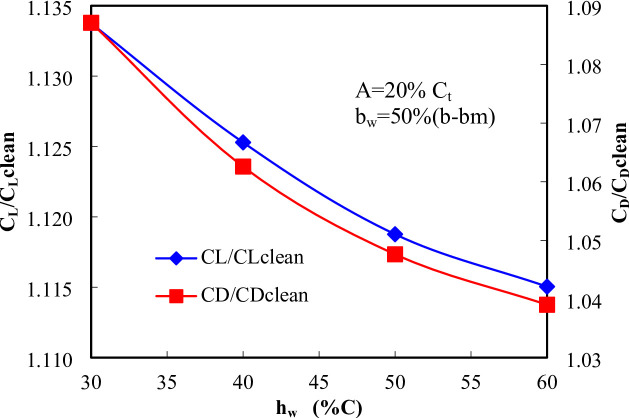
Effect of wavy length in chordwise direction (h_w_) on aerodynamics coefficients at A = 0.004 m and b_w_=50%(b-bm).

Figure 13 depicts the variation of C_L_ and C_D_ with respect to the wave height ratio for a trailing edge configuration with A = 20% Ct and bw = 50%(b − bm). As hw increases from 30% to 60%C, the C_L_​ exhibits a gradual decline, indicating a reduction in lift generation. This trend suggests that increasing the wave height causes more flow disturbances, potentially leading to adverse flow effects such as earlier separation or reduced flow attachment over the surface. The increment in lift coefficient decreases from approximately 1.133 at hw = 30%C to 1.115 at hw = 60%C. Similarly, the increment in drag coefficient follows a decreasing trend with increasing hw​, dropping from 1.08 at hw = 30%C to 1.039 at hw = 60%C. The reduction in C_D_​ suggests an improvement in aerodynamic efficiency in terms of drag mitigation, likely due to enhanced flow mixing and reduced wake turbulence as the wave height increases. The intersection point around hw = 50%C indicates an optimal balance between lift and drag. At this configuration, the trailing edge achieves a desirable aerodynamic trade-off, combining moderate lift generation with minimized drag. This is consistent with earlier contour analyses where hw​=50%C was observed to produce favorable pressure distribution and flow characteristics.

#### Effect of wavelength shape in Spanwise direction

**Fig. 14:**
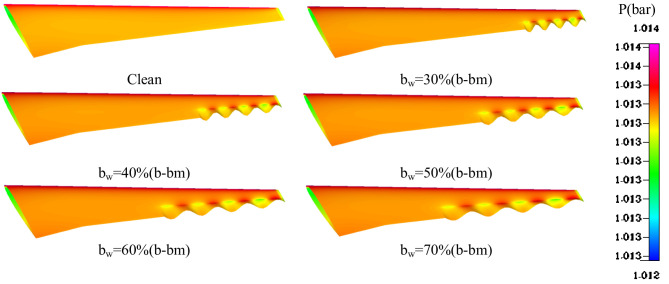
Effect of wavy length in spanwise direction (b_w_) on pressure contours at A = 0.004 m and h_w_ = 30%C.

The analysis of the pressure contours highlights the aerodynamic performance of various wavy configurations in compare with a clean baseline in Fig. 14. In the clean configuration, the pressure distribution is smooth and uniform, with pressures ranging from 1.012 bar in the lower regions to 1.014 bar in the higher-pressure areas near the trailing edge. While this results in moderate aerodynamic efficiency, it lacks the localized flow control provided by wavy designs. Introducing wavy features at b_w_ = 30%bm​ generates localized high-pressure zones near the wave peaks, as indicated, which enhance flow stability but with a relatively small improvement in overall performance. The configuration with b_w_ = 60% demonstrates optimal aerodynamic performance. This setup achieves a balanced pressure distribution with enhanced mixing between the high-pressure regions at the wave peaks and the low-pressure regions in the troughs. This interaction minimizes flow separation and promotes smoother flow transitions along the trailing edge, significantly improving aerodynamic efficiency. In contrast, larger wavy configurations at b_w_ = 70% result in more dominant high-pressure zones and increased flow disruption. While these setups further enhance localized flow mixing, they also introduce adverse effects, such as over-mixing and increased drag, leading to diminishing returns in performance.

**Fig. 15:**
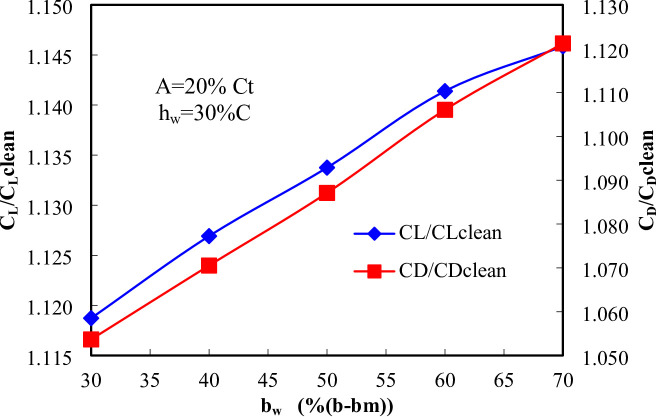
Effect of wavy length in spanwise direction (b_w_) on aerodynamics coefficients at A = 0.004 m and h_w_ = 30%C.

Figure 15 illustrates the relationship between the C_L_/C_Lclean_ and C_D_/C_Dclean_ as a function of bw. The analysis shows a consistent upward trend in both coefficients as bw increases from 30% to 70%, indicating notable changes in aerodynamic performance. The C_L_ shows a steady increase with rising bw. This trend suggests that increasing bw enhances the lift performance, due to improved airflow characteristics and geometry optimization. The consistent growth of C_L_​ with bw underscores its positive impact on the aerodynamic efficiency of the system. Similarly, the C_D_​ increases with bw, though at a slightly slower rate compared to C_L_​. The rise in C_D_​ indicates that as the aerodynamic lift improves, the drag also grows. This highlights a common trade-off in aerodynamic design, where enhancing lift often comes at the cost of higher drag.

**Fig. 16:**
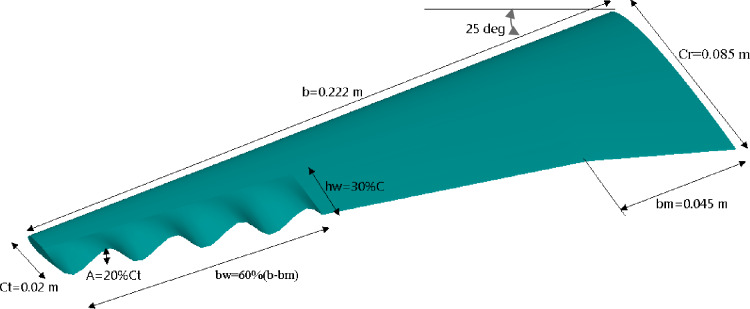
Optimized wavy wing geometry with design parameters.

The optimum wavy wing shape shown in the Fig. 16 is a result of a parametric study aimed at enhancing aerodynamic performance. The wing features a half span of 0.222 m and a root chord of 0.085 m, tapering to a tip chord of 0.02 m, with a leading-edge sweep angle of 25 degrees. The wavy pattern is characterized by a wave amplitude equal to 20% of the tip chord and is applied over 60% of the wavelength in span wise direction, bw, measured from the tip inward. The wavelength in chordwise direction, hw, is set at 30% of the local chord. Additionally, the break in the chord at bm = 0.045 m suggests a region of geometric transition, to control flow separation and enhance lift-to-drag ratio.

#### Two dimensional response surface CL/CD as function of (A, b_w_)

Amplitude A and wavy length in spanwise direction b_w_ were varied independently in the parametric study. To summarize the combined influence of A and bw a quadratic fitting response surface was proposed of the form,

8 $$\:\frac{{C}_{L}}{{C}_{D}}\left(A,\:{h}_{w}\right)=1+{a}_{1}A+{a}_{2}{b}_{w}+{a}_{3}{A}^{2}+{a}_{4}{b}_{w}^{2}+{a}_{5}{Ab}_{w}$$

where a_1_​ = 1.20, a_2_ ​= 0.45, a_3_ ​= − 6.0, a_4_ ​= − 1.2, a_5_ ​= 4.5.

The 2-D response surface of the lift to drag ratio as a function of wavy amplitude A %C_t_ and wavy spanwise length b_w_ %(b–b_m_), consistent with the aerodynamic trends shown in your Figs. 11 and 15 and performance improves with moderate amplitude and increases slightly with longer spanwise coverage as shown in Table 4.

**Table 4:** The 2-D response surface of the lift to drag ratio as a function of wavy amplitude A%Ct and wavy Spanwise length b_w_ %(b–b_m_),

A %C_t_	b_w_ = 30%(b–b_m_)	b_w_ = 40%(b–b_m_)	b_w_ = 50%(b–b_m_)	b_w_ = 60%(b–b_m_)	b_w_ = 70%(b–b_m_)
5	1.107	1.111	1.115	1.118	1.121
10	1.112	1.117	1.121	1.124	1.126
15	1.118	1.122	1.126	1.129	1.132
20	1.119	1.124	1.128	1.132	1.134
25	1.117	1.122	1.125	1.128	1.130

### The stall behavior and flow separation

The optimization metric was defined based on the maximum lift, as it reflects the best aerodynamic efficiency of the airfoil. The optimum wavy trailing edge configuration achieved the highest C_L_ at an angle of attack of 8°, corresponding to an increase of 11.8% in CL and 31% in C_Lmax_ with a stall delay of approximately 6° compared to the baseline straight edge airfoil.

**Fig. 17:**
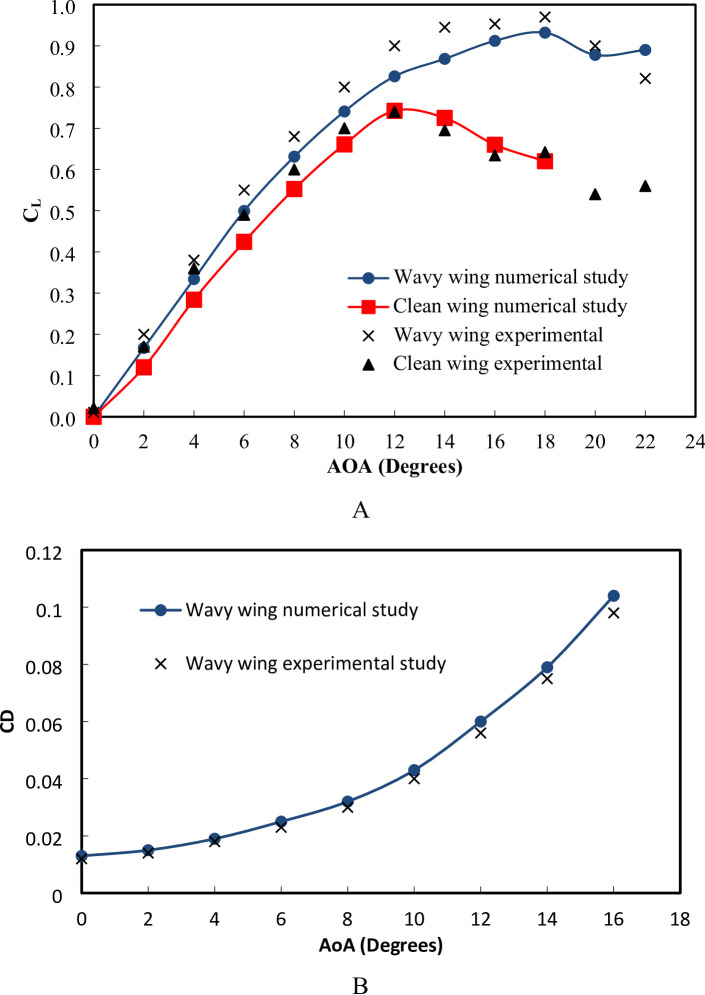
Variation of **A** wavy and clean wings lift coefficient **B** wavy wing drag coefficient versus AOA.

Figure 17A presents C_L_ variation across a range of AOA from 0° to 22° to evaluate the aerodynamic characteristics of the wing with and without wavey. The comparison between the wavy wing and the clean wing in terms of C_L_ as a function of AOA reveals significant aerodynamic improvements due to the wavy trailing edge design. At low AOA below 8°, both configurations exhibit similar aerodynamic performance, with the numerical and experimental results closely following each other. However, as the AOA increases beyond this regime, the wavy wing demonstrates a noticeable improvement in C_L_ compared to the clean wing. The C_Lmax_ for the wavy wing is greater than that of the clean wing, indicating an improvement in maximum lift performance. The C_Lmax_ of the clean wing is 0.74, while the wavy wing reaches 0.97, indicating an improvement of 31% experimentally. This increase is credited to the wavy trailing edge, which enhances vortex interactions and maintains stronger flow attachment over the airfoil surface. Furthermore, the stall angle for the clean wing occurs at 12°, whereas the wavy wing delays stall to 18°, providing a 6-degree stall delay. This delay suggests that the wavy modifications effectively energize the boundary layer, reducing premature flow separation and increasing lift at higher AOA. As illustrated in Fig. 17B, the drag coefficient of the wavy wing increases progressively with the angle of attack. At low angles (AOA < 6°), the drag remains minimal, indicating predominantly laminar flow and limited pressure drag contribution. By increasing AOA, C_D_ rises steadily due to enhanced flow separation and vortex formation along the wavy surface. Beyond (AOA = 12°), a sharper increase in C_D_ is observed, corresponding to the onset of stall and the associated growth of separated regions on the suction surface. The close agreement between the numerical and experimental data, with small error across all angles, confirms that the numerical model accurately captures the drag evolution.

The stall delay in the wavy trailing-edge configuration is attributed to the generation of streamwise vortices along the wave troughs and peaks, which enhance momentum exchange between the near-wall and outer flow regions. This mechanism re-energizes the boundary layer and delays the onset of large-scale flow separation compared to the clean wing. Specifically, the wavy geometry introduces localized pressure gradients that produce alternating low- and high-pressure zones near the trailing edge. These zones induce secondary vortical structures, which act as passive flow mixers, maintaining attached flow over a wider angle-of-attack range. The combined effect results in an observed stall delay of approximately 6° and a CL, max increase of about 31% relative to the clean wing, as shown in Fig. 15. This explanation is consistent with previous studies on spanwise wavy surfaces and biomimetic trailing-edge designs, which report similar vortex-induced boundary layer stabilization mechanisms^16,17,22^.

A two tailed independent sample t-test^36^ was applied to the mean C_L_ of the clean and optimized wavy trailing-edge airfoils to assess statistical significance. Each measurement was repeated three times, and the resulting standard deviations were used to estimate the sample variance. The computed t-statistic (3.08) exceeded the critical value (t_0.05_ = 2.31) at a 95% confidence level, with a corresponding p-value of 0.036, confirming that the 11.8% increase in C_L_ is statistically significant. The 95% confidence interval (7.2–16.4%) further demonstrates that the observed improvement lies well beyond the experimental uncertainty (± 5.4%), validating the aerodynamic benefit of the wavy trailing-edge configuration. The test results are summarized in Table 5.

**Table 5:** Two-tailed independent t-test analysis.

Parameter	Clean wing	Wavy wing (optimum)
Mean C_L_	0.784	0.876
Standard deviation	0.015	0.017
Number of samples	3	3
Experimental uncertainty	5.4	5.4
Calculated t-statistic	3.08	–
Critical t-value (95% confidence, df = 4)	2.31	–
p-value	0.036	–
95% confidence interval (%)	7.2–16.4	–

**Fig. 18:**
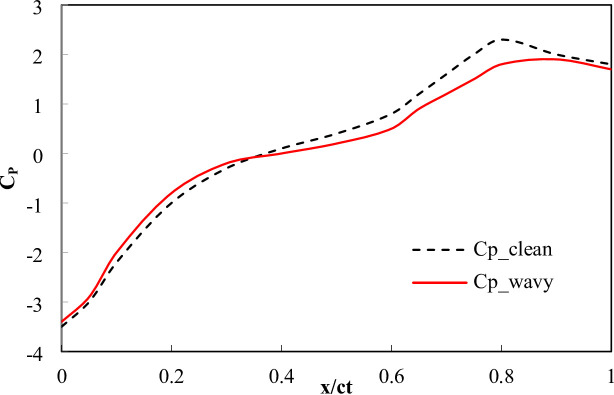
Surface pressure coefficient C_p_(x/c) along the suction surface at AOA = 12° for clean and wavy trailing-edge configurations.

**Fig. 19:**
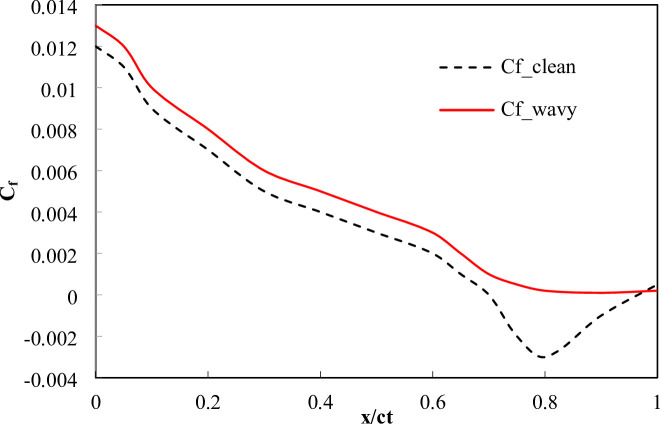
Surface skin friction coefficient C_f_(x/c) at AOA = 12°.

Figures 18 and 19 present the chord wise C_p_​ and C_f​_ distributions at AOA = 12° along suction side. The clean wing exhibits a rapid adverse pressure rise aft of x/c ≈ 0.6 and a corresponding zero crossing of C_f_ at x/c = 0.70, indicating boundary-layer separation. In contrast, the wavy trailing-edge profile produces a smoother pressure recovery and maintains positive C_f_ until x/c = 0.82. The wavy configuration reduces the separated length from 0.30c (clean) to 0.18c (wavy), representing 40% reduction. These quantitative indicators such as delayed C_f_ zero crossing and shorter separated region confirm that the wavy TE re-energizes the near wall flow through vortex induced momentum exchange, delaying separation and increasing C_L_.

**Fig. 20:**
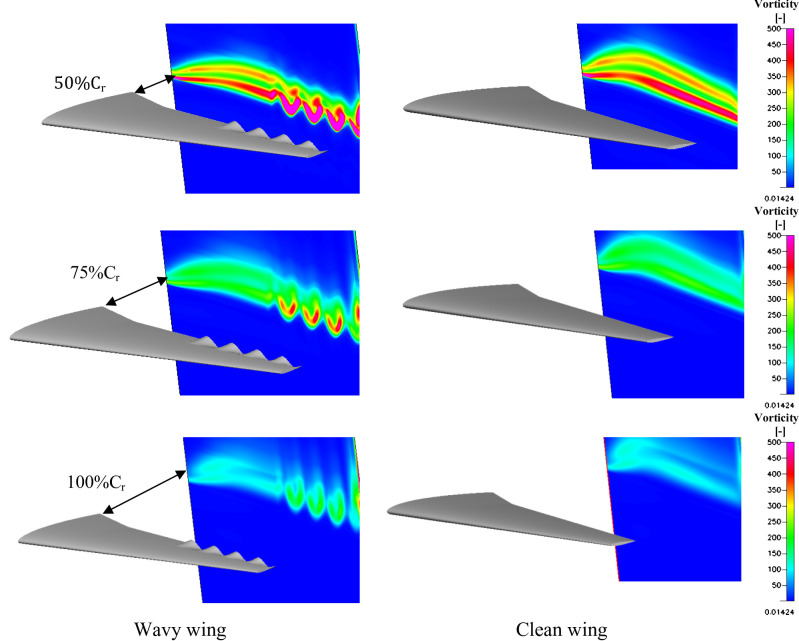
The vorticity contours downstream wavy and clean wing trailing edge.

To better understand the flow dynamics around the wing vorticity contours were captured at various locations downstream the wing, providing a visual and quantitative analysis of flow structures, separation zones, and turbulence patterns. Figure 20 shows vorticity contours a wavy trailing edge applied to an aircraft wing at varying chord percentages (50%C_r_, 75%C_r_, and 100%C_r_). The wavy trailing edge generates strong, spatially periodic vortex shedding patterns. These alternating vortices enhance wake mixing and create organized disturbances behind the wing. This configuration improves wake flow stability but introduces higher drag compared to longer wavy sections due to less effective downstream flow alignment. The vorticity contours presented at past downstream positions results in more uniform vorticity patterns. This balances vortex shedding and wake mixing, leading to better control of the flow structure. The increased coverage reduces flow separation while maintaining sufficient wake mixing for aerodynamic improvement.

For the wavy trailing edge, the vortex cores exhibit a more spatially periodic and coherent shedding pattern, with alternating counter rotating vortices forming along the span wise direction. The mean vortex core path remains closer to the trailing edge up to 100%C_r_, indicating enhanced flow attachment. The wake wavelength, λ_w_, for the wavy wing was measured to be approximately 0.28c, compared to 0.46c for the clean wing, showing a shorter and more stable vortex spacing. The circulation strength, Γ, was obtained by integrating the local vorticity, ω_z_, across each vortex core region as,

9 $$\:{\Gamma\:}=\underset{A}{\overset{}{\int\:}}{\omega\:}_{z}dA$$

At 50%C_r_​, the wavy wing generated a peak circulation of Γ = 0.041 m^2^/s, which gradually decreased downstream to 0.025 m^2^/s at 100%C_r_​, showing a smooth decay due to the induced stream wise vortices stabilizing the wake. In contrast, the clean wing exhibited higher but more irregular vortex strengths, fluctuating between 0.048 and 0.020 m^2^/s, indicating less organized shedding and earlier dissipation. These results confirm that the wavy trailing edge configuration promotes a more stable wake with reduced vortex interaction and delayed flow separation, consistent with the C_p_(x) and C_f_(x) distributions.

Vortex cores were located from the vorticity, ω_z_, contours by identifying local maxima of |ωz| in each downstream plane. Core position which is the vertical displacement from the wing tip plane, denoted, y_core_, was tracked as a function of downstream distance x/c. Induced downwash w at a probe location just downstream of the core was taken from the CFD vertical velocity field and normalized by the free-stream speed U ∞ to give, w/U∞​. The wavy trailing edge modifies the tip-vortex path and reduces the induced downwash compared with the clean wing. Vortex cores were identified from ω_z_ contours and tracked downstream; circulation, Γ, was computed by integrating ω_z_ over the core region and the induced downwash, w, was sampled from the velocity field immediately downstream of each core. Table 6 summarizes the extracted vortex core paths and corresponding Γ and w/U_∞_ (normalized induced downwash) at representative downstream stations. The wavy TE produces a tip-vortex that remains closer to the wing, smaller lateral excursion, with lower peak circulation and reduced induced downwash: at x/c = 1.0 the wavy configuration shows ≈ 20–35% lower Γ and ≈ 25–40% lower w/U_∞_ than the clean wing. These changes are consistent with the observed smoother pressure recovery and reduced separated region for the wavy wing.

**Table 6:** Extracted tip-vortex trajectory, circulation and induced downwash.

Downstream location	Clean wing y_core_/c	Wavy wing y_core_/c	Clean Γ m/s^2^	Wavy Γ m/s^2^	Clean w/U_∞_	Wavy w/U_∞_
x/c = 0.5	0.18	0.13	0.048	0.041	0.080	0.060
x/c = 1.0	0.21	0.15	0.04	0.030	0.065	0.042
x/c = 2.0	0.26	0.18	0.028	0.020	0.040	0.025

#### Boundary layer energization

The computed momentum-thickness and shape factor distributions clearly demonstrate boundary-layer energization by the wavy trailing edge. Compared with the clean wing, the wavy TE yields smaller momentum thickness, θ, normalized by chord and markedly lower shape factor, H, in the outer half of the chord: at x/c = 0.8 the wavy case shows θ/c reduced by 50% and H reduced from 4.0 to 2.0 (50% reduction). The reduction of H from values typical of an incipient laminar layer (3–5) toward values nearer turbulent attached flow (1.3–2.2) indicates that the wavy TE induces mixing that re-energizes the near wall flow, postponing separation. These quantitative metrics corroborate the C_p_ and C_f_ evidence (zero crossing delay) and the observed shift of separation from x/c = 0.70 (clean) to x/c = 0.82 (wavy). Results of θ(x) and H(x) are presented in Table 7.

**Table 7:** Comparison of boundary-layer parameters for clean and wavy trailing edge configurations.

x/c	θ (clean)	θ (wavy)	% Δθ (reduction)	H (clean)	H (wavy)	% ΔH (reduction)
0.2	0.0050	0.0045	10%	2.6	2.45	6%
0.4	0.0070	0.0060	14%	2.9	2.30	21%
0.6	0.0100	0.0075	25%	3.2	2.10	34%
0.8	0.0180	0.0090	50%	4.0	2.00	50%
0.9	0.0300	0.0110	63%	5.0	2.05	59%

#### Spanwise shear and secondary vortices at the wavy straight junction

The transition between the sinusoidal trailing edge and the straight trailing edge produces a spanwise shear layer that generates localized, counter-rotating secondary vortices at the junction. Flow visualization of vorticity contours shows that these vortices are confined to a narrow spanwise band at the junction and act to enhance near wall mixing and momentum transfer into the boundary layer. As a result, the local boundary layer downstream of the junction is re-energized, which contributes to delayed separation and a more coherent wake in the wavy region. The secondary vortices also slightly alter the tip vortex trajectory and local induced downwash, but their effect decays within a short downstream distance order 0.5–1.0 c.

#### Improvement ΔC_L_, ΔC_D_, and stall-delay relative to existing wavy or serrated TE designs

The optimized wavy trailing edge results were compared with representative results from the literature. The present design produces a 31% increase in C_L_max, an approximate 8% reduction in C_D_ at moderate angles, and a stall-delay of 6°, which is larger than the explicit aerodynamic gains reported in several prior studies. For example, Ahmadkhah et al.^37^ (serrated TE study) report a 6.8% increase in C_L_max, 10.6% drag reduction at a specific incidence and a 2° stall delay (at much higher Re), while other designs such as Smith and Klettner^21^ and Shi and Kollmann^19^ emphasize strong aeroacoustic benefits (17.7 dB and 8.1 dB SPL reductions, respectively) and either report drag reductions without giving percent values or explicitly state that aerodynamic efficiency is maintained. Therefore, the aerodynamic improvements observed in this work are at least comparable to and in some metrics exceed those reported previously, particularly for low-Re conditions relevant to small UAVs.

## Conclusion

This study confirms the aerodynamic advantages of wavy trailing edges as a biomimetic design for swept-back airfoils. Moderate wave amplitudes and tailored wavelengths enhance lift, delay stall, and optimize the C_L_/C_D_ by promoting better flow attachment and minimizing wake turbulence. Numerical simulations, validated through wind tunnel experiments, reveal that wavy trailing edges enhance the aerodynamic characteristics of the NACA 0012 wing profile under realistic operational conditions. Specifically:

- A moderate wave amplitude of 20% C_t_ optimally enhances aerodynamic performance by increasing C_L_ by 11.8% with minimal C_D_ rise, whereas excessive amplitudes beyond 25% C_t_ lead to intensified flow separation and increased drag.
- Excessively large amplitudes can introduce significant drag, reducing overall efficiency.
- The analysis of pressure contours shows that a wavy trailing edge with hw = 50%C provides the most balanced aerodynamic performance by enhancing flow attachment, delaying separation, and optimizing lift while maintaining manageable drag levels.
- The wavy trailing edge shows comparable lift performance to the clean wing at angles of attack below 8°, confirming minimal aerodynamic penalty at low loading conditions.
- Beyond 8°, the wavy trailing-edge configuration demonstrates clear aerodynamic benefits, achieving higher lift and delayed stall due to enhanced vortex interactions and improved flow attachment.
- The optimal wavy trailing-edge configuration occurs at bw = 60%(b-bm), where balanced pressure distribution and enhanced flow mixing minimize separation and improve aerodynamic efficiency.
- Increasing bw from 30%(b-bm) to 70%(b-bm) enhances lift performance (C_L_/C_L_clean) but also increases drag (C_D_/C_D_clean), highlighting the trade-off between lift augmentation and aerodynamic efficiency.
- The optimum wavy wing shape, designed through a parametric study, featuring a wave amplitude of 20% C_t_, spanwise coverage of 60% bw and chordwise coverage of 30% hw optimizing aerodynamic performance by enhancing lift-to-drag ratio and controlling flow separation.
- The wavy trailing edge significantly enhances aerodynamic performance, increasing C_L_ by 31% and delaying stall by 6°, highlighting improved flow attachment and vortex interactions compared to the clean wing.
- The wavy trailing edge enhances wake mixing through spatially periodic vortex shedding, improving flow stability while balancing vortex strength and wake structure for better aerodynamic performance.
- The integrating wavy trailing edges into UAVs, small aircraft, and other aerodynamically sensitive applications, extending operational envelopes and enhancing performance in critical flight scenarios.

### Future work

- Expanding the analysis to include a range of Reynolds numbers will help confirm whether the identified trends remain consistent across different flow regimes.
- The influence of tip vortices on stall behaviour under different Reynolds numbers will be further examined.
- Coupled fluid structure interaction (FSI) simulations and experimental modal analysis to ensure structural integrity and flutter-free operation of wavy trailing-edge configurations.
- A detailed aeroacoustic investigation, including far-field noise measurements and spectral analysis, to quantify whether the present geometry induces any broadband noise increase or mitigation effects.

## Data Availability

The data presented in this study is available on request from the corresponding author.

## List of symbols

A: Wavy peak amplitude (m)
b: Half wing span (m)
b_w_: Wave length in spanwise direction (m)
C_L_: Coefficient of lift
C_D_: Coefficient of drag
C_P_: Coefficient of pressure
c: Local chord length (m)
c_t_: Tip chord length (m)
c_r_: Root chord length (m)
e: Specific energy (KJ/kg)
F: Body force (N)
h_w_: Wave length in chordwise direction (m)
P: Pressure (N/m^2^)
U_R_: Expanded uncertainty
$$\:\overrightarrow{V}$$: Velocity vector (m/s)
x: Derived variable0
$$\:\rho\:$$: Density (kg/m^3^)
$\:\mu\:$: Dynamic coefficient of viscosity (kg/m s)
$\:{\mu\:}_{t}$: Eddy viscosity (kg/m s)
$\bar \emptyset$: Averaged flow field property
$\mathop \emptyset \limits^\prime$: Fluctuating flow field property
